# In situ flip osteotomy bone block: an alternative bone graft harvesting site proposal for anterior maxillary bone defects. A case report at 3-year follow-up

**DOI:** 10.1007/s10006-025-01498-1

**Published:** 2026-01-09

**Authors:** Davide Farronato, Leonardo Romano, Luca Poncia, Lorenzo Azzi, Luca Levrini, Marco Farronato

**Affiliations:** 1 Department of Medicine and Technological Innovation, School of Dentistry, University of Insubria, Varese, Lombardy 21100 Italy; 2 Department of Medicine and Surgery, School of Dentistry, University of Insubria, Varese, Lombardy 21100 Italy; 3 Department of Human Sciences, Innovation and Territory, School of Dentistry, Postgraduate of Orthodontics, University of Insubria, Varese, Lombardy 21100 Italy; 4 Ospedale Maggiore Policlinico, Fondazione IRCCS Cà Granda, Milan, Lombardy 20122 Italy

**Keywords:** Dentistry, Implantology, Vertical bone regeneration, Onlay graft, Piezoelectric surgery.

## Abstract

**Background:**

This article describes a novel approach for the reconstruction of vertical and horizontal bone defects in the anterior maxilla that involves the use of an autogenous block graft harvested and rotated from the same surgical site. A case with a 3-year follow-up, featuring a vertical-horizontal bone deficiency, is presented as an example.

**Case presentation:**

A bone deficiency is presented at the 2.2 position. Through the use of a piezoelectric instrument, a bone block apical to the defect is harvested. The osteotomy produces a preoperatively planned volume and shape of the bone block, which is then perfected, flipped upside down and fixed using osteosynthesis screws, with the thickest portion in the crestal area. The reconstruction provided sufficient volume for the implant rehabilitation. After 3.5 years, bone remodelling appears contained; the horizontal volume is maintained.

**Conclusions:**

When residual anatomy appears eligible for this approach, this technique may represent an alternative to ectopic bone harvesting, thus reducing the patient’s discomfort and surgical invasiveness.

## Introduction

As described by Cawood and Howell in 1988 [1], the alveolar process undergoes morphological changes after tooth loss. Wolff’s law explains these modifications, affirming that bone mass and structure adapt to the mechanical loads to which they are subjected [2]. Since the load needed to maintain bone volume can not be reached after tooth loss, a decrease in the latter will occur [3].

In case of insufficient bone quantity, bone augmentation procedures may be necessary to obtain a correct three-dimensional implant position for successful aesthetic and functional outcomes and long-term survival of implant-prosthetic rehabilitation [4, 5]. Several treatment options for vertical bone defects are described in the literature. These include guided bone regeneration (GBR) that can be performed using titanium-reinforced non-resorbable e-PTFE membranes [6, 7], resorbable collagen membranes without any fixation [8], resorbable collagen membranes fixated with tenting screws [9], titanium mesh covered by resorbable collagen membranes [10], and resorbable collagen membranes fixed with osteosynthesis plates [11]. Other options are bone block grafts and alveolar distraction osteogenesis [12].

Three main strategies for bone block grafting are presented in the literature: the onlay, the inlay and the shell technique [13]. The onlay block graft is the most conventional approach and consists in placing a bone block over the defect; then the graft is fixed with titanium screws, and the gaps between the block and the recipient site are filled with bone chips [14]. The inlay or sandwich technique involves the execution of a horizontal cut in the alveolar bone 10 mm below the crest. A segment of the alveolar ridge is then mobilised with two vertical cuts and is moved coronally, while the space created is filled by the interposition of a bone block [15]. In the split bone block (SBB) technique, described by Khoury et al. [16], also known in the literature as the shell technique, a bone block is divided into two parts of about 1 mm thickness, which are placed on the defect to create a “container” that is filled with autogenous particulated bone.

Exploring literature dealing with block harvesting in the same surgical site that has to be reconstructed, these are the highlighted contributions found.

Anitua et al. [17] first described in 2014 the use of an in situ bone block graft for the treatment of a horizontal defect of the upper jaw, in conjunction with sinus floor augmentation, in 11 patients. In particular, the graft was harvested from the lateral wall of the maxillary sinus and applied to the labial surface of the alveolar process to increase its width. The gaps between the graft and recipient site were filled with plasma rich in growth factors (PRGF), and all was covered with a PRGF membrane. At 5 months of follow-up, the CBCT analysis showed a mean horizontal bone gain of 5.4 mm.

In 2016, T. Wang et al. [18] published one case of horizontal and vertical alveolar process reconstruction in the anterior maxilla using two in situ bone blocks. These were harvested below the nasal base with a rectangular shape. Then, one was secured above the alveolar ridge and the other on the labial surface of the alveolar process. A certain amount of inorganic bovine particulated bone was used to fill the residual volume. Therefore, a titanium mesh and a concentrated growth factor (CGF) membrane were positioned to cover all. Four months later, no significant resorption was found at the CBCT examination, and an adequate bone volume for implant placement was achieved.

Afterwards, J. Wang et al. [19] published a cohort study, conducted on a sample of 24 patients, that compares the results obtained in the treatment of horizontal bone defects in the anterior maxilla with traditional ectopic and in situ onlay block grafts, associated with GBR. The in situ grafts were harvested apical to the recipient site with a rectangular shape, and in both groups, the realisation of the osteotomy lines, as well as the repositioning of the graft, were guided by the use of a surgical template. At a mean follow-up period of 1.7 years, the measurements performed through the superimposition of the CBCT scans demonstrated no differences in terms of success rate, complications, and horizontal width changes between the two groups. Furthermore, the in situ onlay block group showed better vertical stability.

Yang et al. [20] described a technique that consists in using an in situ onlay autogenous bone block graft in patients with horizontal ridge deficiencies. In his description, the harvested block was never shaped according to the volume deficiency, but harvested with trephine drills, thus round. The block was then repositioned and stabilised with a single screw to increase the horizontal width of the alveolar process. The grafts were associated with the use of particulate bone and covered with a collagen resorbable membrane.

Yuan et al. [21] proposed a similar technique, called “in-situ bone ring technique”, that consists in harvesting a bone block with a round shape. The main difference with Yang et al. [19] is that the recipient site was decorticated by making a circular groove. In both articles, the CBCT examination performed 6 or more months after the surgical procedure showed a satisfactory horizontal bone gain and maintenance, according to the authors.

In 2018, Altay et al. [22] introduced a novel technique for reconstruction of the severely resorbed posterior maxillae where the tuberosity bone was harvested, flipped, and reimplanted in the nearby surgical site, to reconstruct the defect vertically and horizontally. The residual gaps between the graft and the alveolar bone were filled with alloplastic particulated bone. This method was applied to 3 patients, for a total of 5 grafts, and the radiographic examination at 3 years of follow-up evidenced an acceptable resorption rate.

As an alternative to these techniques, the present article aims to show a novel approach called the “flip osteotomy”, applied in the anterior maxilla. The rationale consists in harvesting the basal bone in the same area of the defect, flipping its orientation upside down to have the widest and thickest portion on the coronal aspect. A geometrical evaluation before the surgery is applied to check the possibility of creating a block shape and volume according to the final volume requirements, and its fine adaptation to the recipient site. A clinical example is described, where this approach is applied. The main purpose is the restoration of a vertical and horizontal defect without the involvement of a second surgical site.

## Case presentation

### Technique description

**Fig. 1:**
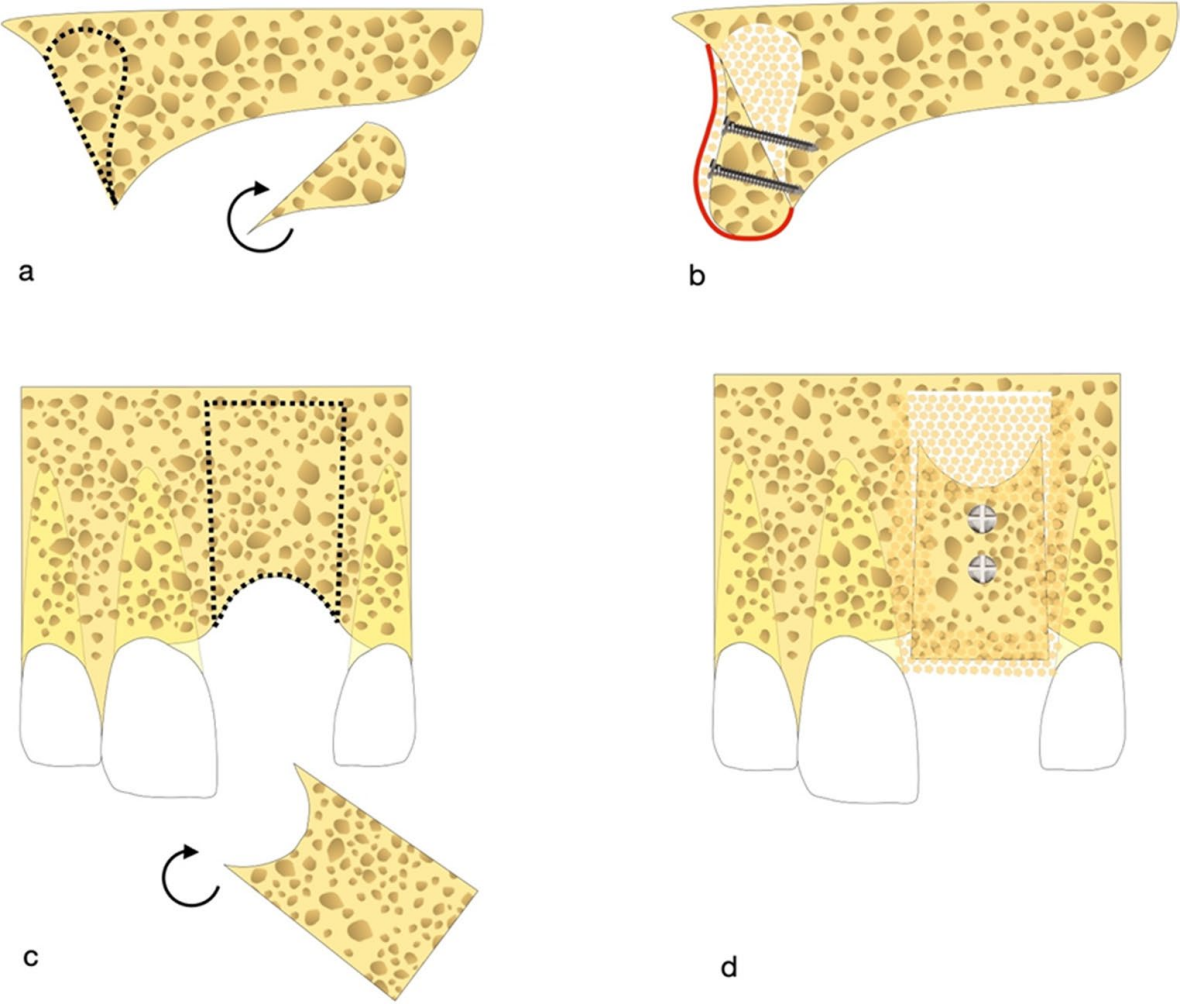
Schematic description of the flip osteotomy in sagittal (**a**, **b**) and frontal perspective (**c**, **d**). A bone block is harvested from the same site to be reconstructed, rotated and fixed with osteosinthesis screws. The residual spaces are filled with particulated bone and all is covered with a resorbable membrane

The flip osteotomy (Fig. 1) consists in harvesting a bone block graft from the basal bone apical to the area that has to be reconstructed.

The design of the bone block is achieved by two divergent cuts in the apical direction, which widen as much as necessary to be able to reconstruct the defect, extending up to 2 mm below the piriformis fossa. The osteotomy lines are performed with a piezo-electric instrument, respecting the roots of the adjacent elements. On the sagittal plane, the cuts proceed in a palatal direction to obtain a transverse thickness that matches the extension of the defect to be restored. Horizontally, the osteotomy proceeds until the cortical of the base of the nose is identified. Therefore, after beginning the section of the crestal cortical with a piezo-electric instrument, the final separation of the bone block is completed with osteotomy chisels.

The bony ridge of the piriformis fossa must be maintained so as not to alter the anatomy of the face profile and the support of the perinasal tissues.

After smoothing the bone roughness in the region of the sampling, the bone block is modelled, using diamond burs and bone scrapers, to adapt it to the shape of the defect. Then the block is flipped apico-coronally and fixed in the site with osteosynthesis screws to the residual palatal bone. In this way, the thickest portion of the block is in the crestal area, to regenerate the defect both vertically and horizontally. The block’s apical portion is kept distant from the residual bone to create a shell-like condition, similarly to Khoury’s described technique [16].

### Case example

**Fig. 2:**
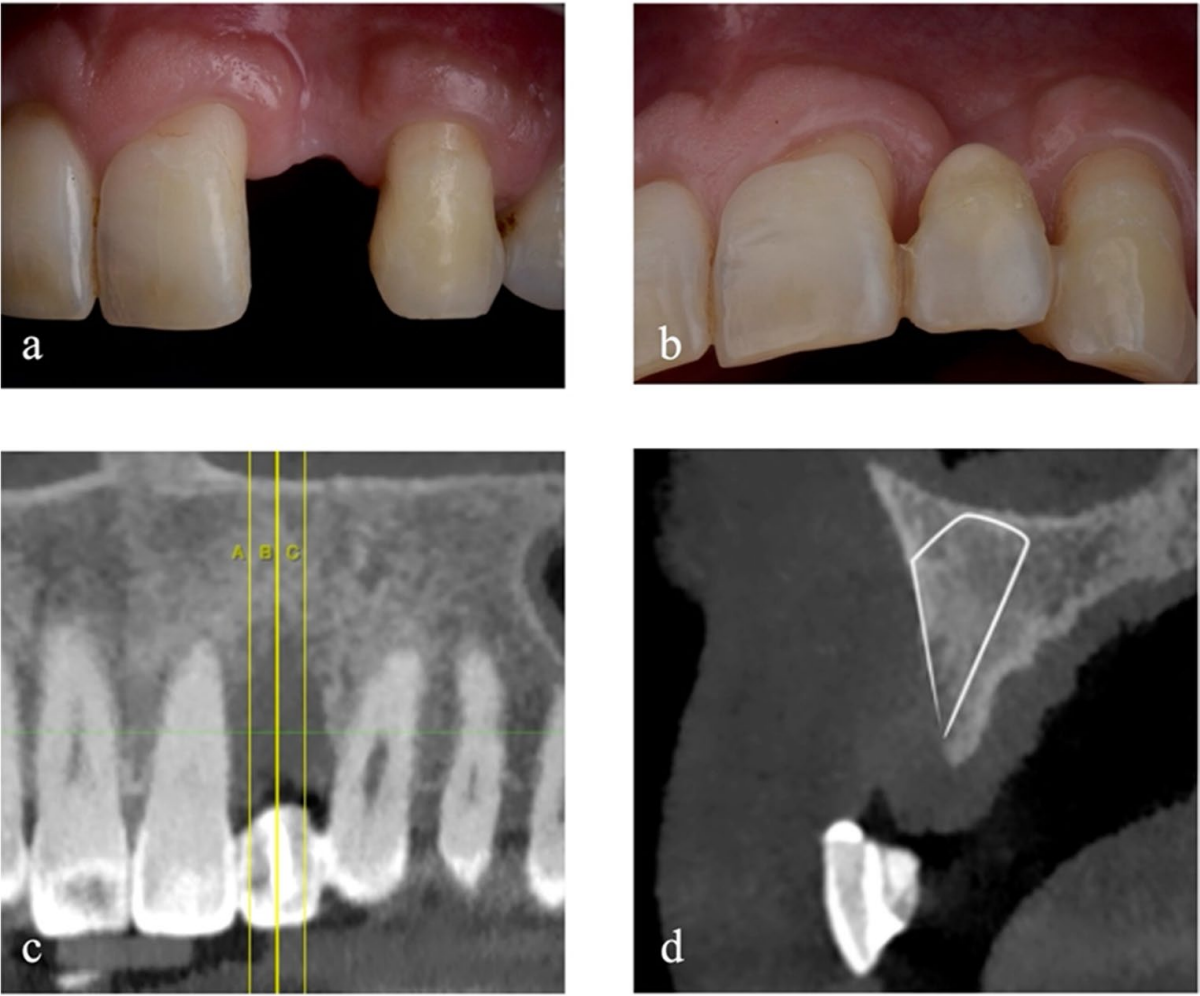
Baseline view in the region of element 2.2

The intraoral examination reveals the presence of bone and keratinised tissue deficiencies at the edentulous saddle corresponding to the position of 2.2 (Fig. 2a and b). Moreover, the adjacent teeth developed gingival recessions due to the interdental bone peak loss.

At the CBCT examination, the distance from the remaining bone peaks to the cement-enamel junction (CEJ) is measured mesially and distally on the palatal and buccal side of the edentulous area. The defect axially measures 9.1 mm mesially and 6.6 mm distally on the vestibular side. Palatally, instead, the defect is less pronounced, with a bone deficiency of 3.2 mm and 2.0 mm, respectively, mesially and distally, from the CEJ of the adjacent teeth. It is possible to identify a satisfactory amount of bone volume in the area confined between the alveolar crest and the base of the nasal cavity, and the roots of the adjacent teeth (Fig. 2c and d).

Therefore, the choice is to reposition the basal bone of the surgical site to minimise the invasiveness of the treatment.

A full-thickness flap is designed to identify the site of interest between the midline and the distal part of the homolateral premolar root. Afterwards, the cortical bone is exposed, and the roots of the adjacent teeth are cleaned with mechanical instruments.

Once the nasal base is identified, a block is set up according to the principles previously described, without damaging the roots of 2.1 and 2.3, and preserving the integrity of the choanae and its tissue support. The block is removed and its shape is adapted to the recipient site. Afterwards, the graft is flipped and fixed on the recipient site by using two osteosynthesis screws.

The superficial gaps between the bone block and the alveolar bone are filled by autologous bone chips, resulting from the adaptation process. A fine coating of low-resorbable-rate heterologous chips is then spread on the block’s buccal exposed surface and in the apical space-making residual defect. A resorbable pericardium membrane covers everything.

**Fig. 3:**
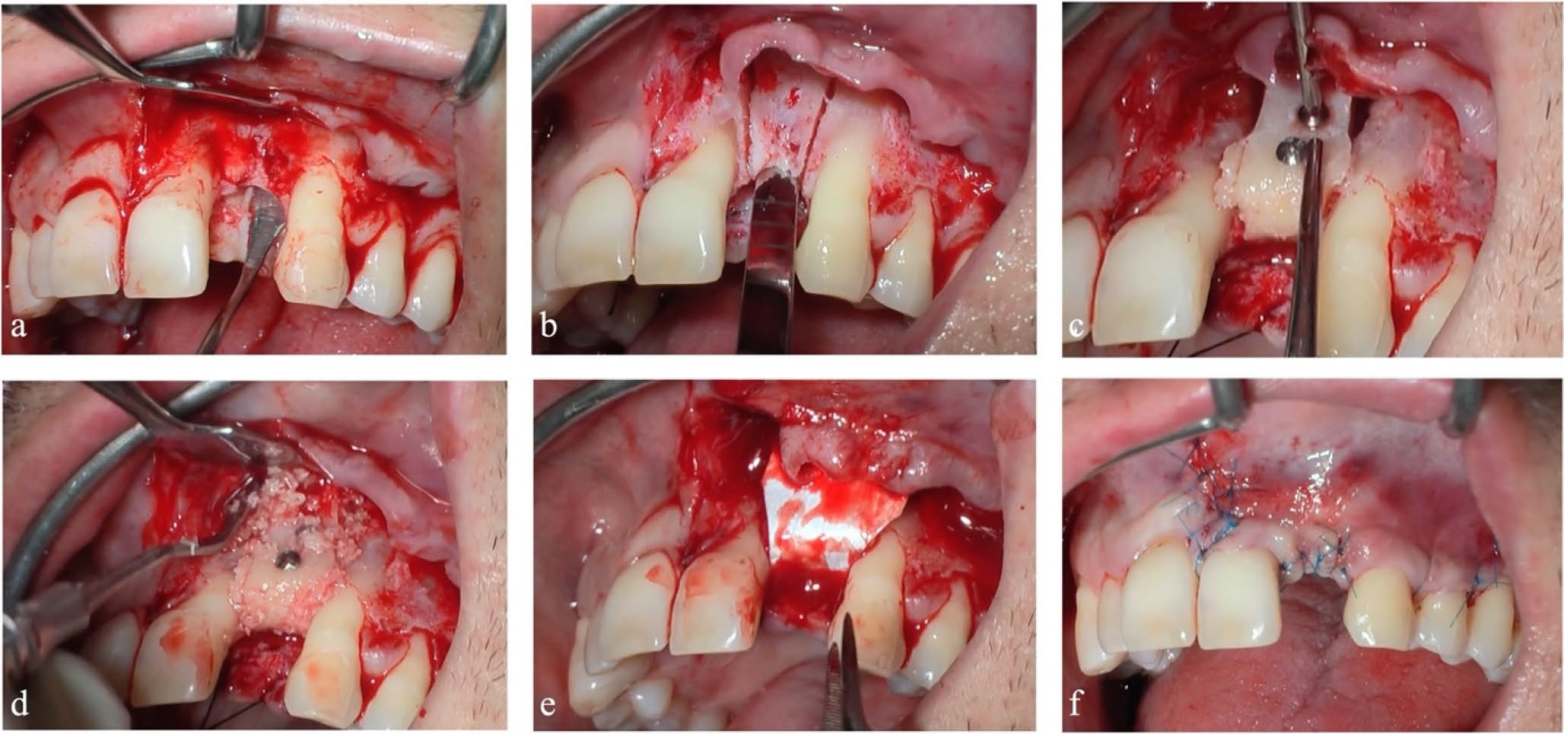
Bone block graft procedure. Full-thickness flap elevation (**a**). Osteotomy lines for the mobilization of the bone block graft (**b**). Bone graft fixed in the recipient site (**c**). Gaps between bone block graft and the recipient site filled with particulated bone (**d**). Graft protection with resorbable membrane in pericardium (**e**). Primary intention suture of the flaps (**f**)

Periosteal release incisions are performed to achieve flap passivation and to ensure a tension-free suture. A set of single and horizontal mattress sutures is applied, thus reducing the risk of surgical wound dehiscence (Fig. 3).

**Fig. 4:**
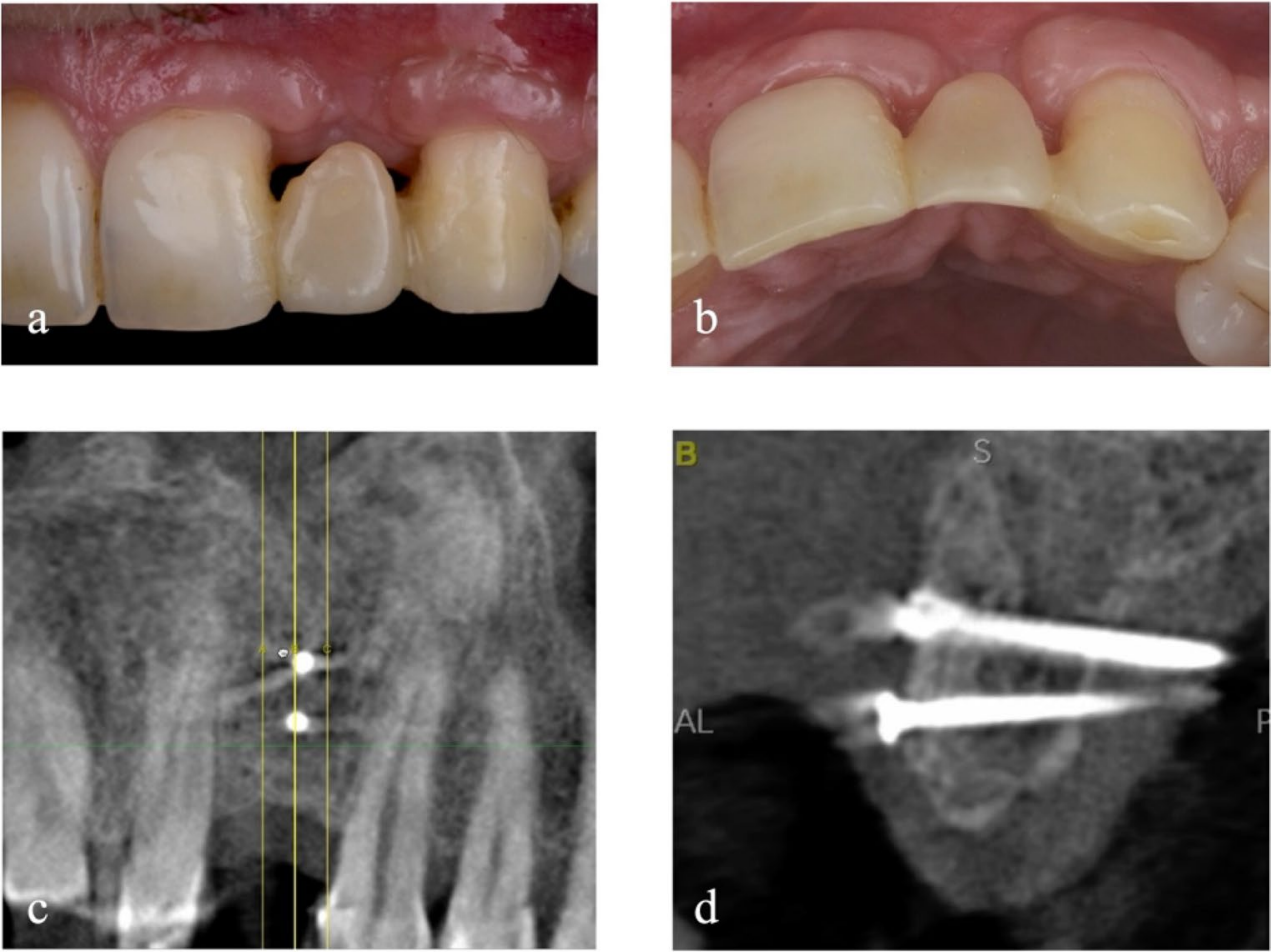
Detail of soft tissue (**a**, **b**) and CBCT examination of the reconstructed refect (**c**, **d**) 6 months after grafting procedure

Two weeks after surgery, sutures are removed. After 6 months from the placement of the bone graft, the examination and radiographic investigations are performed to evaluate the integration of the grafted bone and the soft tissue integrity (Fig. 4).

**Fig. 5:**
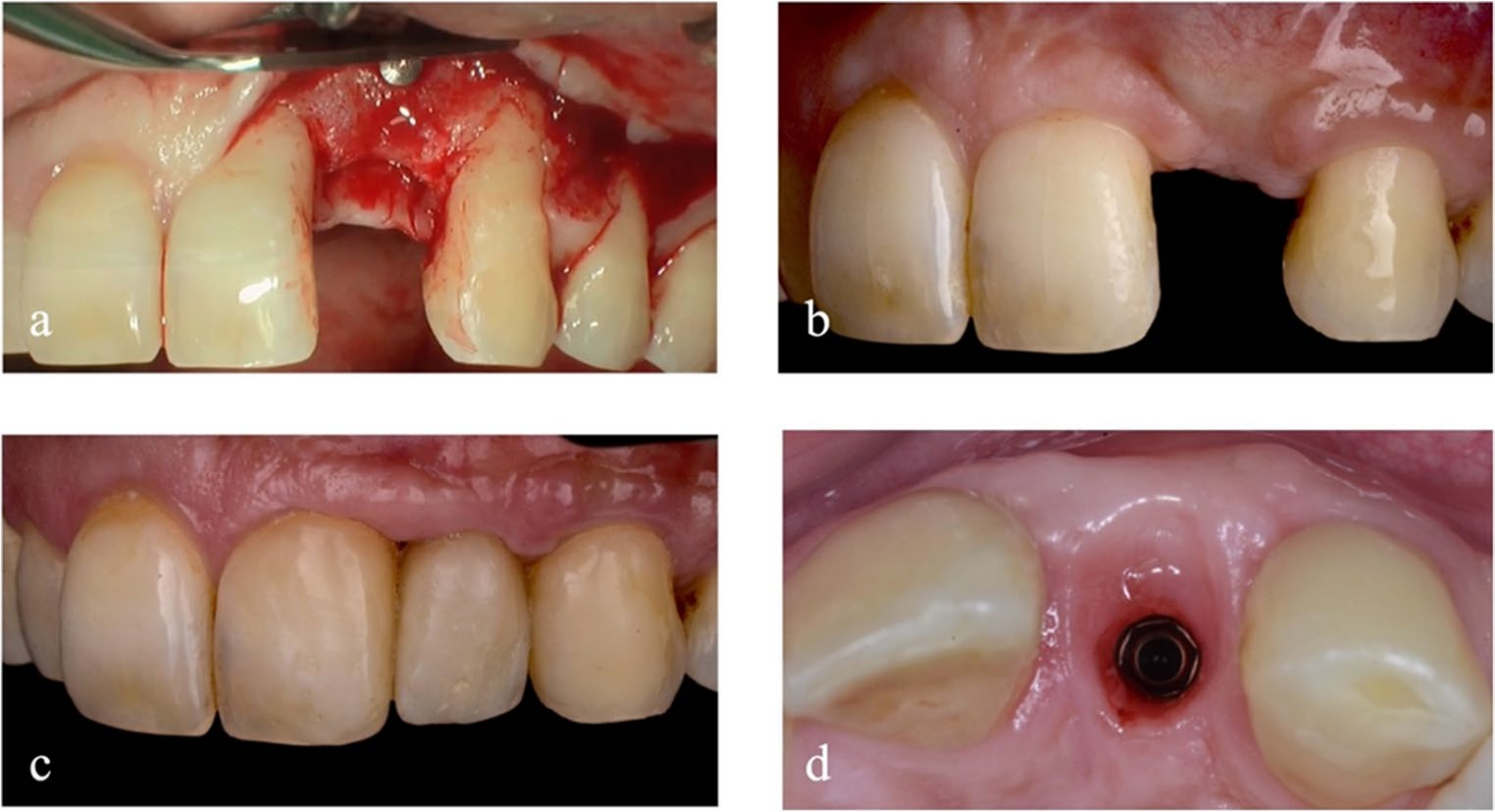
Detail of reconstructed site before implant placement (**a**) and 4 months after, before soft tissue augmentation (**b**). Detail of soft tissues 6 months after provisional placement (**c**, **d**)

The surgical site is reopened at 6 months by setting up a full-thickness mucoperiosteal flap to remove the fixation screws (Fig. 5a). At the same time, an implant (Anyridge^®^, MegaGen, Gyeongbuk, Republic of Korea) with a 3.8 mm diameter and 10 mm length is placed.

After another 4 months (Fig. 5b), soft tissue augmentation techniques are performed through a connective tissue graft harvested from the palate. The implant is uncovered another 2 months later, and a temporary screw-retained crown in PMMA is delivered to condition the tissues and obtain the most biomimetic result possible.

The condition is reevaluated 1.5 years after the bone block grafting procedure. The root exposure is reduced on the contiguous element, but the mesial embrasure presents a black triangle. This is managed through an apicalization of the prosthetic contact point. Six months after the provisional delivery (Fig. 5c and d), the definitive prosthesis is positioned: a zirconia-ceramic screw retained crown.

**Fig. 6:**
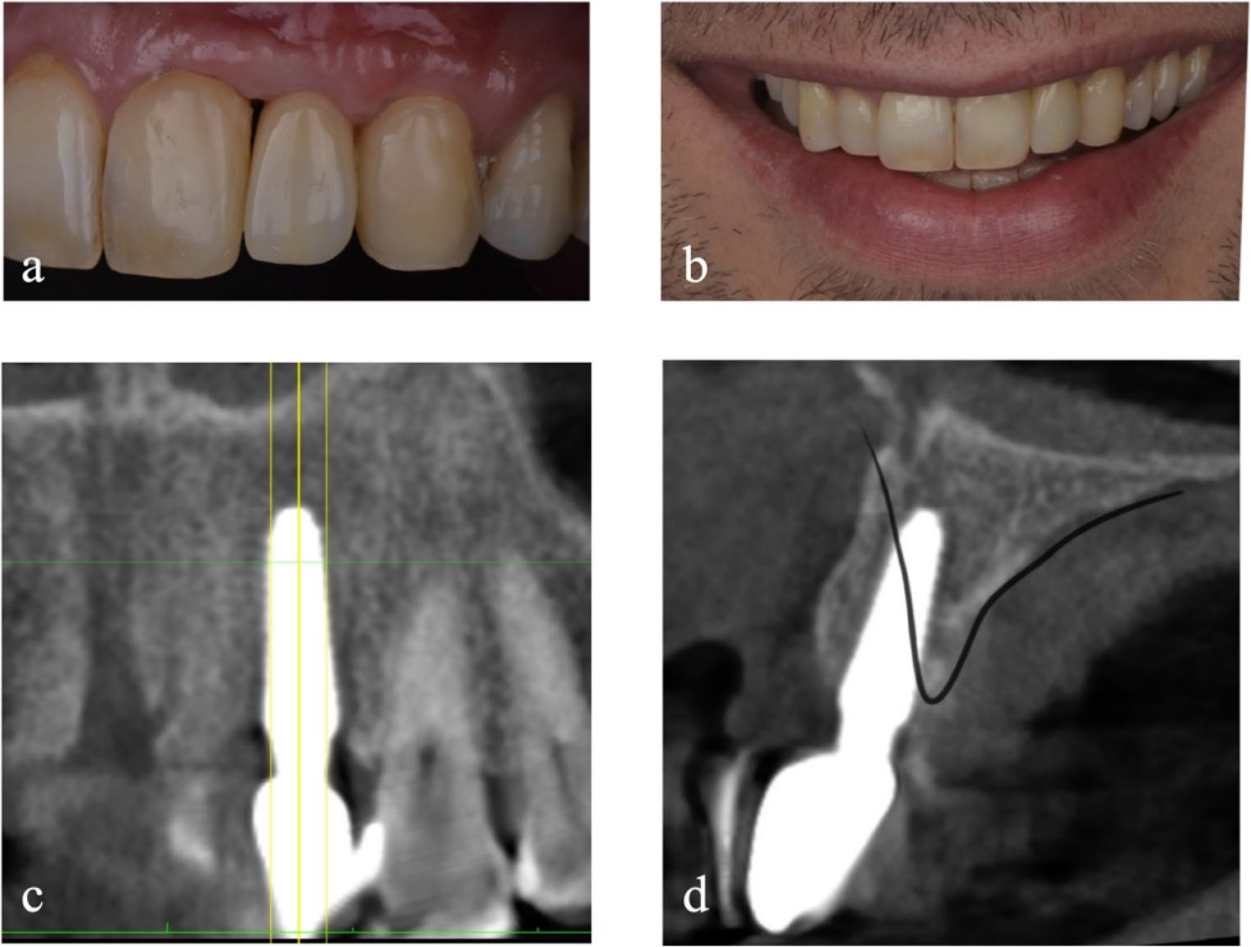
Intraoral (**a**) and extraoral (**b**) outcome of the rehabilitation at 3.5 years after the first surgery, with CBCT control (**c**, **d**)

At 3.5 years after bone block grafting, a CBCT control examination is performed (Fig. 6c and d). The approach used shows sufficient horizontal bone stability, while some remodelling occurred in the mesial peak region, not exceeding 2.60 mm. The distal peak, on the other hand, appears stable.

Physical examination shows a reduction in the degree of recession of the gingival margin of the elements adjacent to the defect. Finally, although the filling of the mesial gingival embrasure is incomplete, the distal interproximal papilla is better represented, and the patient’s aesthetic needs are met (Fig. 6a, b). The initial mean between the mesial and distal vertical distance at T0 from the bone peak to the CEJ (BP-CEJ MEAN M-D) on the vestibular side was 7.85 mm, while on the palatal side 2.60 mm. After 6 months of healing from the vertical regeneration, and at 3.5 years of follow-up, the BP-CEJ MEAN M-D on the vestibular side registered 3.01 mm and 3.22 mm, respectively. Palatally, this parameter remained almost stable, registering 3.11 mm and 2.63 mm. Therefore, the mean vertical bone gain was 4.63 mm buccally (Table 1).

**Table 1:** Note: This data is mandatory. Please provide

	BP-CEJ MV	BP-CEJ DV	BP-CEJ MP	BP-CEJ DP	BP-CEJ MEAN MV-DV	BP-CEJ MEAN MP-DP
T0	9.10	6.60	3.20	2.00	7.85	2.60
6 months	2.88	3.14	3.22	2.99	3.01	3.11
3.5 years	3.61	2.83	2.92	2.33	3.22	2.63

Bone peak - CEJ distance at T0 (before grafting), 6 months and 3.5 years measured at mesio-vestibular (MV), disto-vestibular (DV), mesio-palatal (MP) and disto-palatal (DP) sides

**Fig. 7:**
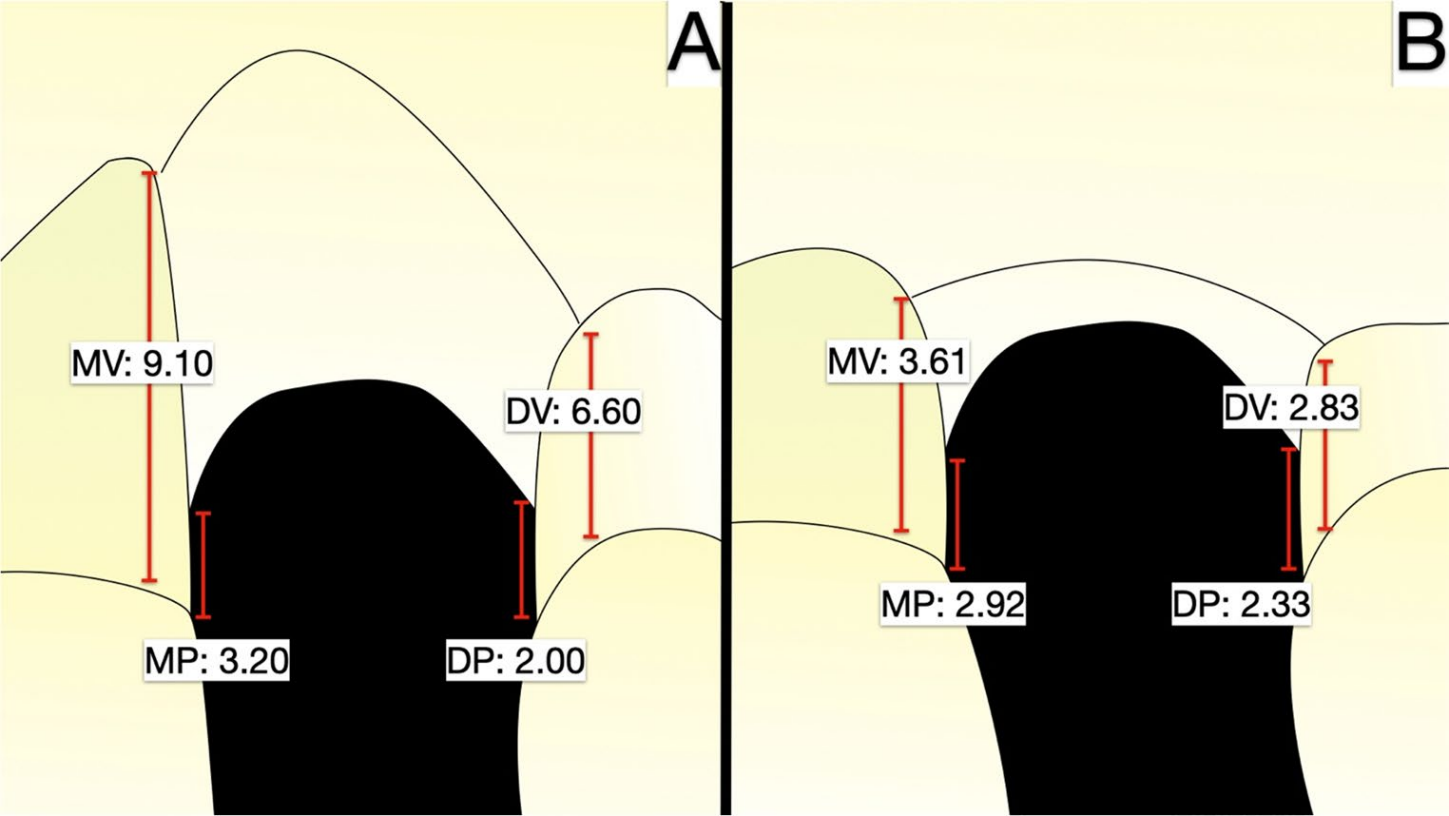
(**A**) Measurements between the mesio-vestibular, disto-vestibular, mesio-palatal, and disto-palatal peaks and the CEJ of the neighboring teeth at T0. The measurements were taken three dimensionally on the CBCT, and repeated on the follow-up CBCT at 3.5 years in the same way (**B**)

In the picture below (Fig. 7) it is reported schematically the measurements taken in four points between the bone peaks and the CEJ before the procedure and after the regeneration at 3.5 years of follow-up.

## Conclusions

This article has shown an alternative technique for the treatment of vertical bone defects in the anterior maxilla, which consists in using an autogenous block graft harvested from the same surgical site and then rotated before its fixation.

Among all the alveolar bone reconstruction procedures, the use of autogenous bone grafts still represents a gold standard for most of the authors [23], especially in vertical defects with complicated morphology [12, 13]. This is because no other material has all the same biological properties, which include osteoinduction, osteoconduction, and osteogenicity [24, 25].

Autogenous bone block grafts used for the treatment of vertical defects have a success rate that exceeds 95% and early implant failure is equal to 0.38% within 3 years of placement [26]. However, the use of autogenous bone block grafts is associated with a significant resorption rate, between 25% and 60% [26]. Nevertheless, this can be reduced using an organic bone layer on top of the exposed surfaces [27]. The graft can also be covered with a membrane to provide better dimensional stability [28], as done in the clinical case described in this paper.

In terms of volume stability, the onlay technique seems to be the most subject to resorption over time [29]. The inlay or sandwich technique is thought to guarantee higher stability than the onlay one [29], but to obtain a more stable result, the shell technique is currently considered the first choice [13] because of a resorption rate equal to 11.4% after 10 years [30]. Urban et al. [31] in a systematic review and meta-analysis established that the mean vertical bone gain that can be achieved with different autogenous bone block graft techniques, measured from the CEJ of the adjacent teeth to the base of the bone, is 4.12 mm (with 95% confidence interval 3.11–5.13 mm). This agrees with the results obtained in this article. The greater bone gain seems to be seen using the shell technique [13], as demonstrated by a study conducted by Khoury et al. [30] that shows a mean vertical bone gain of 6.72 ± 2.26 mm at 10 years of follow-up.

The distance between the interproximal bone peak (BP) and contact point (CP) is one of the most relevant parameters that influences the presence of a complete papilla. Tarnow et al. found that a complete filling of the interdental space through the papilla, in natural teeth, can be achieved in 100% of the cases if the BP-CP is less than 5 mm [32]. Considering implants, a threshold can not be established [33], but a maximum of 5 mm seems to be recommended [34]. This explains the appearance of a black triangle in the presented case on the mesial side at 3.5 years of follow-up, where the interproximal bone resorption increased the BP-CEJ distance, thus, as a consequence, the BP-CP measure.

However, in addition to efficacy, the choice of surgical technique must also consider the needs of the patient: the acceptance of the procedure should be high, while the morbidity of the procedure should be minimal [26]. With ectopic autogenous bone blocks, post-operative morbidity may be higher if compared to other techniques, mostly related to the need for a second surgical site [36]. Possible complications include nerve injuries with permanent or temporary paresthesia, pulp necrosis, graft exposure, infections, pain, swelling, secondary haemorrhage, and incomplete integration of the bone block [13, 26, 35]. Furthermore, bone block grafting procedures are time-consuming and technically demanding [36]. In this perspective, the technique proposed, which is based on the use of a bone block harvested from the same site where the defect is present, avoids access to a second surgical site. Therefore, the possibility of post-operative complications, the time required for the surgery and the technical difficulty can be significantly lower, while still obtaining a satisfactory result. The alternative to reduce patient discomfort is the use of xenogenic bone block grafts [37], but limited data are available [38].

Beyond this article, some other authors described the use of in situ bone blocks [17–22], but these techniques differ from that proposed in this paper in some aspects, which include the site and type of defect that is reconstructed, the shape of the graft and/or its orientation. Some of these studies refer to horizontal defects in the anterior or posterior maxilla [17, 19–21], so the obtained results are not comparable with those of the present article. Other studies [18, 22] performed vertical reconstructions, but did not report any measurements.

The methods applied by Yang et al. [20] and Yuan et al. [21] are based on the harvesting of round-shaped bone grafts. Given the geometry of the harvested block, the orientation can not be considered fundamental, which differs from the proposal of the flip osteotomy. Furthermore, the round shape prevents the use of the block when attempting to apply it to interproximal bone peaks or adapt it to complex geometries. Another potential limitation is the need to use a wide amount of particulated material, which, due to the absence of a rigid scaffold, might redistribute to unwanted shaping, similarly to the sausage technique inspected limitations [39].

Among the discussed in situ techniques, the only other one that involves the use of a flipped block is the one described by Altay et al. [22]. Nevertheless, it is limited to a distal upper sector in the absence of an adjacent tooth and does not relate to an intercalated bone deficiency, where the presence of missing bone peaks and the dental roots may augment the complexity of the surgical intervention. Furthermore, if the third molar is present, the tuberosity can not be harvested, while in the anterior region, it is more likely to find a sufficient bone volume. It should also be emphasised that the quality of the bone obtained is different from that of the anterior region because the tuberosity is less dense. However, this procedure may still be considered suitable for bone reconstruction when specific conditions of the patient are met.

The in situ flip osteotomy block for the reconstruction of vertical bone defects in the anterior maxilla may be considered a novel approach proposed by the authors of this article. According to the results obtained, it could be an alternative to other bone block grafting methods, reducing the morbidity related to the need for a second surgical site.

Potential limitations of this approach could be found in the surgeon’s experience or the presence of the nasopalatine nerve in the surgical site, which could reduce the volume available for the harvesting of the block or even prevent the use of this treatment option. Moreover, the use of surgical templates for the realisation of the osteotomy lines should be recommended for a more precise and safe procedure [40]. The flip osteotomy seems indicated for intercalated defects that affect the space of one or two elements, while wider defects may not be geometrically suitable.

As another limitation, the linear measurements of the vertical defect executed in this study can only be useful as a rough guide to understand the anatomy before and after the regeneration, since they were performed on a single patient. Further verifications are needed, with long-term randomised clinical trials supported by consistent data, before this technique may be recommended in daily clinical practice.

The removal of the block from a site adjacent to the graft recipient one allowed a reduction in operating invasiveness and discomfort for the patient. Considering the strategy of a spatial reorientation and an appropriate design of the osteotomy, it may expand the treatment options for bone deficiencies. The article is limited to the description of a novel approach through a single example; thus, further inspection is recommended before considering the technique safe and reliable.

## Data Availability

No datasets were generated or analysed during the current study.

## Abbreviations

BP: bone peak
CBCT: Cone-beam computed tomograhpy
CEJ: Cementoenamel junction
CGF: Concentrated growth factor
CP: contact point
DP: disto-palatal
DV: disto-vestibular
e-PTFE: Expanded polytetrafluoroethylene
GBR: Guided bone regeneration
MP: mesio-palatal
DP: disto-palatal
PMMA: Polymethyl methacrylate
PRGF: Plasma rich in growth factors
SBB: split bone block
